# ASO Author Reflections: Which Technique is Superior for Segmentectomy of Small Pulmonary Nodules: Robot-Assisted or Video-Assisted?

**DOI:** 10.1245/s10434-023-13180-5

**Published:** 2023-02-10

**Authors:** Mu-Zi Yang, Hao-Xian Yang

**Affiliations:** 1 Department of Thoracic Surgery, Sun Yat-sen University Cancer Center, Guangzhou City, Guangdong Province People’s Republic of China; 2 State Key Laboratory of Oncology in South China, Collaborative Innovation Center for Cancer Medicine, Sun Yat-sen University Cancer Center, Guangzhou City, Guangdong Province People’s Republic of China

## Past

Segmentectomy has become a new standard of care for patients with selected peripheral-type early-stage non-small cell lung cancer (NSCLC).^1^ Previous study had demonstrated that video-assisted segmentectomy (VAS) as a minimally invasive approach is safe and effective for anatomic segmentectomy.^2^ But the deep learning curve due to the rigid chopstick-like instruments and the two-dimensional vision has hindered its application. In recent years, robot-assisted segmentectomy (RAS) has rapidly increased due to the advantages of three-dimensional vision and articulated mechanic surgical arms. However, the outcome comparison between RAS and VAS for small pulmonary nodules remains insufficient.^3,4^

## Present

This study performed a head-to-head comparison between RAS and VAS for small pulmonary nodules in terms of short-term outcomes. Propensity score-matching (PSM) was performed to minimize selection bias between the groups. The results suggested that the patients undergoing RAS had a shorter total operative time, less blood loss, less use of strong opioids, and a shorter postoperative stay than those undergoing VAS, indicating that RAS is a safer and more effective surgical approach than VAS in segmentectomy of small pulmonary nodules.^5^ Based on these findings, the authors propose that RAS will be more widely used in the treatment of peripheral early-stage NSCLC as the cost becomes lower in the future.

## Future

Nevertheless, the data of this study were from a single center, and the data of the patients in the VAS group were retrospectively collected. Although PSM was performed in this study, some confounding factors that might have had an impact on the outcomes still existed. Therefore, more data from other centers are needed to confirm the results.
